# Enhanced spatiotemporal skeleton modeling: integrating part-joint attention with dynamic graph convolution

**DOI:** 10.1038/s41598-025-18520-x

**Published:** 2025-10-06

**Authors:** Yanghong Qin, Shengrui Liu, Chunhong Yuan

**Affiliations:** 1 School of Artificial Intelligence, Chongqing Three Gorges Vocational College, Chongqing, China; 2 School of Computer and Artificial Intelligence, Southwest Minzu University, Chengdu, 610041 China; 3 Faculty of Control Systems and Robotics, ITMO University, St. Petersburg, Russia 197101

**Keywords:** Patiotemporal skeleton modeling, Part-joint attention, Dynamic graph convolutional network, Human motion prediction, Action recognition, Computational biology and bioinformatics, Engineering, Mathematics and computing, Neuroscience

## Abstract

Human motion prediction and action recognition are critical tasks in computer vision and human-computer interaction, supporting applications in surveillance, robotics, and behavioral analysis. However, effectively capturing the fine-grained semantics and dynamic spatiotemporal dependencies of human skeleton movements remains challenging due to the complexity of coordinated joint and part-level interactions over time. To address these issues, we propose a spatiotemporal skeleton modeling framework that integrates a Part-Joint Attention (PJA) mechanism with a Dynamic Graph Convolutional Network (Dynamic GCN). The proposed framework first employs a multi-granularity sequence encoding module to extract joint-level motion details and part-level semantics, enabling rich feature representations. The PJA module adaptively highlights critical joints and body parts across temporal sequences, enhancing the model’s focus on salient regions while maintaining temporal coherence. Additionally, the Dynamic GCN dynamically constructs and updates inter-joint spatial relationships based on temporal feature similarities, facilitating effective spatiotemporal reasoning. Extensive experiments on the Human3.6M dataset demonstrate that our method consistently outperforms strong baselines across various prediction horizons. Specifically, it achieves a Mean Per Joint Position Error (MPJPE) of 10.2 mm at 80 ms and 57.5 mm at 400 ms, outperforming the best baseline by 9–12 percentage relative improvement across diverse actions. These results indicate the proposed method’s ability to accurately capture both subtle and large-scale human motions while maintaining temporal stability. This work advances the development of interpretable and precise skeleton-based motion modeling and can benefit broader domains such as real-time human-robot interaction, intelligent surveillance, and behavior recognition in practical environments.

## Introduction

Human motion prediction is a critical task in computer vision and human-computer interaction, enabling intelligent systems to anticipate future human poses based on observed skeleton sequences. This capability supports real-time applications such as intelligent surveillance, human-robot collaboration, and virtual reality^1,2^. While closely related, action recognition focuses on identifying predefined action categories, whereas motion prediction aims to forecast the fine-grained evolution of human movement over time. In this study, we focus specifically on the challenge of human motion prediction using skeleton-based representations, with the goal of capturing dynamic spatiotemporal dependencies and subtle joint-level variations across future time frames.

Traditional approaches to human motion analysis often rely on handcrafted features or simplistic spatiotemporal models, which are limited in capturing the complex dependencies, hierarchical structures, and high-level semantics inherent in human skeleton movements^3^. The emergence of deep learning, particularly convolutional neural networks (CNNs) and recurrent neural networks (RNNs), has significantly advanced the field by enabling effective extraction of temporal features from sequential skeleton data, leading to improved performance in capturing local temporal dynamics and global motion patterns^4^. However, these models still encounter challenges in modeling fine-grained part-level semantics and dynamic spatial dependencies across joints, which are crucial for understanding subtle and context-dependent human actions and transitions.

Graph convolutional networks (GCNs) have further expanded the modeling capacity for skeleton-based action recognition by enabling non-Euclidean structure representation of human skeletons, effectively modeling the spatial topology among joints^5^. Building upon these advancements, the integration of attention mechanisms within spatiotemporal models has shown promise in improving interpretability and precision by enabling the dynamic focusing on salient joints, parts, and time frames within motion sequences^6^. Despite these developments, existing skeleton motion modeling methods continue to face several limitations.

Firstly, many current methods either focus solely on joint-level dynamics or on part-level semantics, lacking effective mechanisms to integrate these complementary perspectives into a unified modeling framework^7^. Secondly, conventional GCN frameworks typically utilize static adjacency matrices to encode spatial relationships between skeleton joints, which restrict their ability to adapt to dynamically evolving configurations during complex human actions^8^. Thirdly, the underutilization of temporal and spatial attention mechanisms limits the models’ capacity to highlight critical body parts and joints adaptively across time, reducing their ability to capture nuanced and context-specific motion patterns necessary for fine-grained human action recognition and motion prediction.

To address these limitations, this study proposes a novel spatiotemporal skeleton modeling framework that integrates the following key components:

1. *A multi-granularity sequence encoding module* that jointly captures local joint-level motion details and global part-level semantic information, enabling the model to represent skeleton dynamics with high fidelity.
2. *A Part-Joint Attention mechanism* that adaptively emphasizes important joints and body parts across temporal sequences, enhancing the model’s focus on salient features while maintaining temporal coherence^9^.
3. *A Dynamic Graph Convolutional Network* that dynamically constructs and updates spatial relationships between joints based on temporal feature similarities, facilitating adaptive and effective spatiotemporal reasoning within human skeleton data.

By systematically integrating these components, the proposed framework effectively models the temporal evolution and spatial topology of human skeleton sequences, leading to improved accuracy and robustness in human motion prediction and action recognition tasks. This work not only advances the methodological landscape of skeleton-based human motion modeling but also lays a solid foundation for practical deployment in intelligent surveillance, behavior analysis, human-robot collaboration, and adaptive learning systems where precise, interpretable, and real-time motion understanding is imperative.

## Related work

Human motion prediction^10^ and action recognition have been extensively studied within computer vision and human-computer interaction, serving as fundamental tasks for enabling intelligent systems to understand and anticipate human behaviors in various real-world scenarios^11^. Early approaches primarily relied on handcrafted features and conventional machine learning methods, which often lacked the capacity to capture the complex spatiotemporal dependencies and hierarchical structures inherent in human skeleton motion data^12^.

The advent of deep learning has significantly advanced the field, with convolutional neural networks (CNNs) and recurrent neural networks (RNNs) demonstrating the capability to learn temporal dependencies from skeleton sequences and improve the modeling of local temporal patterns and global motion trends^13^. However, these methods often struggle with effectively capturing the non-Euclidean spatial topology of human skeletons, which is critical for recognizing nuanced human actions.

To address these challenges, graph convolutional networks (GCNs) have emerged as powerful tools for modeling the structured relationships within skeleton data^5^. Methods such as ST-GCN introduced spatial and temporal graph convolutions to jointly capture spatial dependencies among joints and temporal dynamics across frames, providing a foundational framework for graph-based skeleton modeling^14^. Subsequent studies have advanced this direction by incorporating adaptive graph construction and attention mechanisms within GCN frameworks to enhance flexibility and expressiveness in spatial modeling, allowing dynamic adjustment of joint and edge importance based on motion context^15^.

Despite these advancements, existing GCN-based methods often rely on static adjacency matrices to model spatial relationships, which inherently limits their ability to adapt to dynamically evolving joint configurations in complex human actions^16^. This limitation is particularly pronounced in scenarios involving occlusions, subtle gestures, and partial-body movements, where fixed graph structures fail to capture the fine-grained, context-dependent changes in skeleton connectivity^17,18^.

Several approaches have attempted to enhance spatial reasoning using learnable adjacency matrices or adaptive graph construction techniques. For example, adaptive ST-GCN variants^19^ learn connectivity structures from data, while attention-based GCNs^20^ selectively focus on salient joints or temporal frames. However, these methods often focus solely on either spatial or temporal domains and do not effectively capture spatiotemporal dynamics in a unified manner, limiting their performance in modeling complex motion sequences.

Moreover, few studies have thoroughly addressed the necessity of multi-granularity feature representation, which is essential for integrating fine-grained joint-level details with part-level semantic structures^21,22^. Existing methods frequently lack mechanisms to combine these perspectives, leading to bottlenecks in recognizing subtle and interleaved actions, particularly in real-world settings such as human-robot interaction, intelligent surveillance, and online learning engagement analysis^23^.

Recent works have demonstrated the advantages of modeling coordinated body regions through part-based decomposition. For instance, APL-GCN introduces adaptive part-level embedding for robust skeleton-based one-shot recognition under data-scarce conditions^24^. JP-GA further enhances regional semantic learning by grouping joints and applying group-level attention^25^. STSD decomposes skeleton sequences into semantic parts and applies spatial-temporal transformers for fine-grained recognition^26^. Additionally, Part-Level Knowledge Distillation^27^ improves recognition under occlusions and noise through part-level knowledge distillation, reinforcing the effectiveness of region-aware supervision. These approaches validate the necessity of combining joint-level detail with part-level abstraction in building robust skeleton-based action recognition systems.

In comparison, our proposed method introduces a Part-Joint Attention (PJA) mechanism coupled with a Dynamic Graph Convolutional Network (DGCN) within a multi-granularity encoding framework, addressing several critical gaps in the current literature.Specifically, unlike traditional static GCN approaches, our method dynamically constructs and updates spatial relationships among joints based on temporal feature similarities, allowing the network to adapt to evolving skeleton configurations during motion sequences^28^. This dynamic adaptation enhances the model’s ability to capture complex, fine-grained, and context-dependent skeleton movements.

Furthermore, while previous attention-based models often treat spatial and temporal attention separately, our PJA mechanism jointly emphasizes critical joints and body parts across temporal sequences, facilitating coherent and interpretable spatiotemporal modeling. This design enables the model to leverage both localized joint-level precision and global part-level coordination, which is often overlooked in prior work.

Inspired by hierarchical human body kinematics^29^, our framework effectively combines multi-granularity feature encoding with dynamic spatiotemporal reasoning, leading to enhanced robustness and interpretability in human motion prediction and skeleton-based action recognition^6,30,31^. Compared to existing methods, the proposed approach offers a unified, adaptable, and interpretable framework that addresses the limitations of static graph structures and isolated attention mechanisms, thereby advancing the state of the art in skeleton-based human motion modeling.

## Methods

### Overall structure

**Fig. 1:**
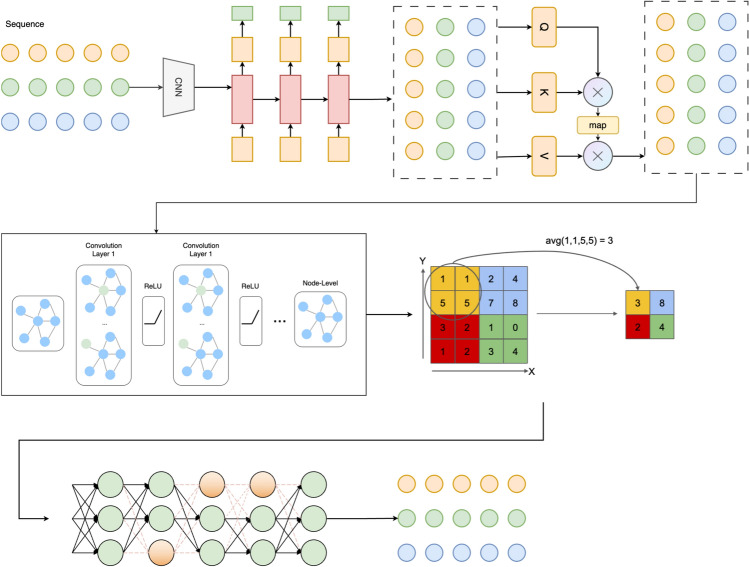
Overall structure of the proposed framework.

To effectively model the spatiotemporal dependencies within human skeleton sequences, this paper proposes a framework that integrates a Part-Joint Attention mechanism with a Dynamic Graph Convolutional Network (Dynamic GCN). The proposed architecture captures high-level semantic correlations across temporal steps and structural hierarchies in human actions. As illustrated in Fig. 1, the model consists of four modules: sequence feature encoding, attention-enhanced representation learning, dynamic graph-based modeling, and feature aggregation with classification output.

The model takes as input a sequence of human skeleton frames composed of multiple keypoints, structured into joint-level and part-level representations. Each frame is first processed by a one-dimensional convolutional neural network (1D CNN) to extract local temporal features and standardize feature dimensions. Stacked convolutional layers suppress local noise and enhance region-specific sensitivity.

Based on the encoded features, a dual-path Part-Joint Attention module performs parallel temporal modeling over the two structural levels. Through query-key-value triplets, the attention mechanism generates weighted temporal maps that enhance the original feature sequences, allowing the model to focus explicitly on critical body parts and salient time points to capture subtle motions and part-level synergies.

To further incorporate spatial structural information, the attention-refined features are passed into a dynamically constructed graph. A multi-layer graph convolutional network (GCN) is employed to model spatial relationships between skeleton nodes. During the graph construction phase, edge connections are dynamically updated based on temporal feature similarities, allowing for adaptive neighborhood aggregation. Each GCN layer fuses features from neighboring nodes and applies nonlinear activation functions (e.g., ReLU) to increase representational capacity. This design enhances the model’s ability to encode dynamic dependencies and structural coupling across body parts.

Finally, the node-level embeddings produced by the GCN are compressed via a global pooling layer and passed through a fully connected classifier to produce action category or status predictions. Through the collaborative integration of convolutional encoding, attention-based refinement, and graph-structured reasoning, the proposed model demonstrates superior ability in capturing the temporal evolution and spatial topology of skeleton data, making it well-suited for tasks such as action recognition, behavior classification, and engagement estimation.

### Multi-granularity sequence encoding module

To effectively capture fine-grained motion dynamics and part-level semantic structures within skeleton sequences, we design a multi-granularity sequence encoding module that preserves the topological structure of the human body while extracting rich temporal context features. This module serves as the first stage in our framework, providing structured and comprehensive representations that support the subsequent attention and dynamic graph modeling components.

Let the input skeleton sequence be defined as:

1 $$\begin{aligned} X \in \mathbb {R}^{T \times N \times C}, \end{aligned}$$

where *T* denotes the number of frames, *N* represents the number of joints, and *C* is the feature dimensionality (e.g., 3D coordinates, velocity, or acceleration).

#### Joint-level encoding

To capture localized motion dynamics at the individual joint level, we apply a one-dimensional convolutional neural network (1D CNN) along the temporal dimension for each joint. This operation captures local temporal patterns, such as micro-movements and transitional dynamics, critical for fine-grained motion recognition:

2 $$\begin{aligned} H_j = \textrm{ReLU}(\textrm{Conv1D}(X_j)), \end{aligned}$$

where $X_j \in \mathbb {R}^{T \times C}$ denotes the temporal sequence for joint *j*. The ReLU activation enhances non-linearity and aids in learning effective temporal representations for localized joint motion.

#### Part-level encoding

To encode the semantic information of coordinated body regions (e.g., arms, legs, torso), we define a set of anatomical parts $P_p$, each consisting of a subset of related joints. For each part, we aggregate the encoded joint features using pooling operations:

3 $$\begin{aligned} H_p = \textrm{Pooling}_{j \in P_p}(H_j), \end{aligned}$$

where pooling can be either average pooling (to capture overall movement trends) or max pooling (to highlight the most salient movements) within the part, ensuring that the spatial context and part-level semantics are preserved while reducing sensitivity to noisy joints.

#### Contribution to the overall framework

The multi-granularity sequence encoding module enriches the framework by:

- Providing a structured representation that jointly captures fine-grained joint-level dynamics and high-level part semantics, enabling the system to recognize both subtle and complex movements.
- Serving as a robust input to the Part-Joint Attention module, facilitating adaptive focus on critical joints and parts across time while maintaining temporal coherence.
- Supporting the Dynamic Graph Convolutional Network by supplying multi-scale features that improve spatial reasoning and allow dynamic graph updates based on temporally encoded patterns.

Through this design, the module effectively bridges low-level temporal feature extraction and high-level spatiotemporal modeling, significantly contributing to the system’s ability to achieve high accuracy in human motion prediction and action recognition tasks.

**Fig. 2:**
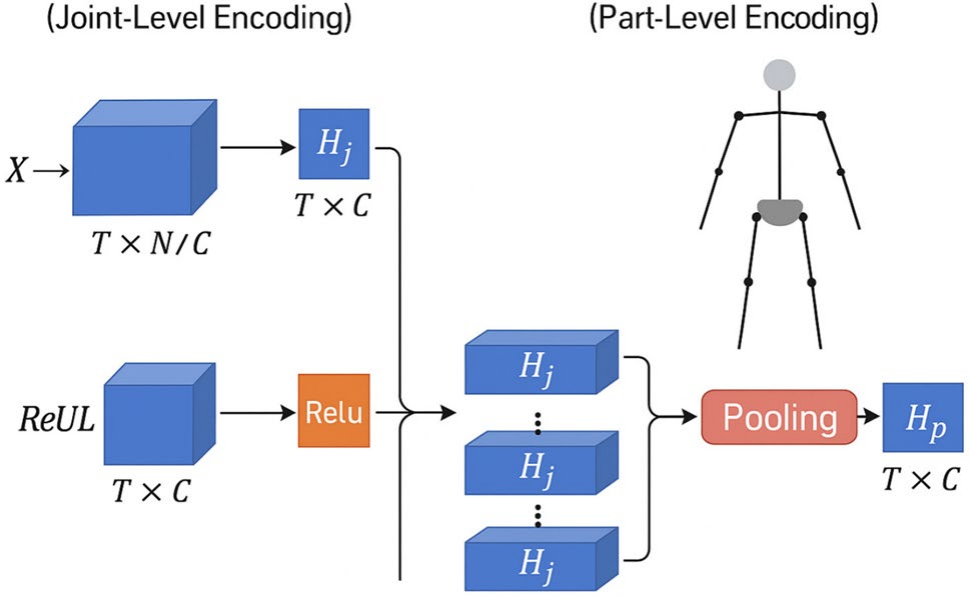
Architecture of the proposed multi-granularity sequence encoding module, illustrating joint-level temporal convolution and part-level semantic pooling for structured feature extraction.

The architecture of the proposed multi-granularity sequence encoding module is illustrated in Fig. 2, showing the joint-level temporal convolution and part-level pooling processes used for constructing the integrated feature representations.

### Part-joint attention module

To dynamically emphasize informative joints and coherent body parts while preserving temporal coherence, we introduce a Part-Joint Attention (PJA) module, which enables the model to adaptively focus on critical skeleton structures over time. This module effectively bridges the multi-granularity sequence encoding stage and the dynamic graph convolutional modeling stage, ensuring that salient joint-level and part-level features are prioritized during spatiotemporal reasoning.

#### Purpose and role

While the multi-granularity encoding module captures fine-grained joint-level dynamics and part-level semantics, these features may contain redundant or noisy signals, especially in scenarios with occlusions or minor postural fluctuations. The PJA module addresses this by:

- *Dynamically weighting* joints and parts based on their contextual importance at each time step.
- *Enhancing robustness* to noisy or occluded keypoints by emphasizing reliable structures.
- *Facilitating semantic interpretability* by explicitly modeling attention across hierarchical body representations.

#### Attention score computation

Given the encoded joint-level features $H_j$ and part-level features $H_p$, the module computes soft attention scores for both streams using scaled dot-product attention:

4 $$\begin{aligned} \alpha _{t,j} = \frac{\exp (q_t^\top k_j)}{\sum _{j'} \exp (q_t^\top k_{j'})}, \quad \beta _{t,p} = \frac{\exp (q_t^\top k_p)}{\sum _{p'} \exp (q_t^\top k_{p'})}, \end{aligned}$$

where queries and keys are defined as:

5 $$\begin{aligned} q_t = W_q h_t, \quad k_j = W_k h_j, \quad k_p = W_k h_p, \end{aligned}$$

where $W_q$ and $W_k$ are learnable weight matrices, and $h_t$ represents the temporal context vector derived from the encoded features.

#### Attention-based feature aggregation

The PJA module then aggregates the weighted features to form an enhanced representation at each time step:

6 $$\begin{aligned} \tilde{H}_t = \sum _j \alpha _{t,j} h_j + \sum _p \beta _{t,p} h_p. \end{aligned}$$

This dual-stream attention aggregation allows the network to capture localized motion cues from individual joints while simultaneously leveraging the global spatial semantics from body parts, thereby improving the model’s understanding of coordinated and contextually relevant movements.

#### Contribution to overall performance

The PJA module contributes to the overall framework in the following ways:

- It ensures that the most informative joints and parts are emphasized during subsequent graph convolution, improving the quality of spatiotemporal feature learning.
- It enables context-aware feature enhancement, allowing the model to adapt its focus dynamically based on action context, which is crucial for fine-grained action recognition and motion prediction.
- It maintains temporal coherence and semantic consistency, providing clear interpretability regarding which body structures influence the model’s decision at each frame.

The PJA module is lightweight and fully differentiable, allowing seamless integration into the end-to-end learning pipeline without incurring significant computational overhead. Its design ensures that hierarchical attention over both joint-level and part-level features directly contributes to the improved accuracy, robustness, and interpretability of the proposed spatiotemporal skeleton modeling framework.

**Fig. 3:**
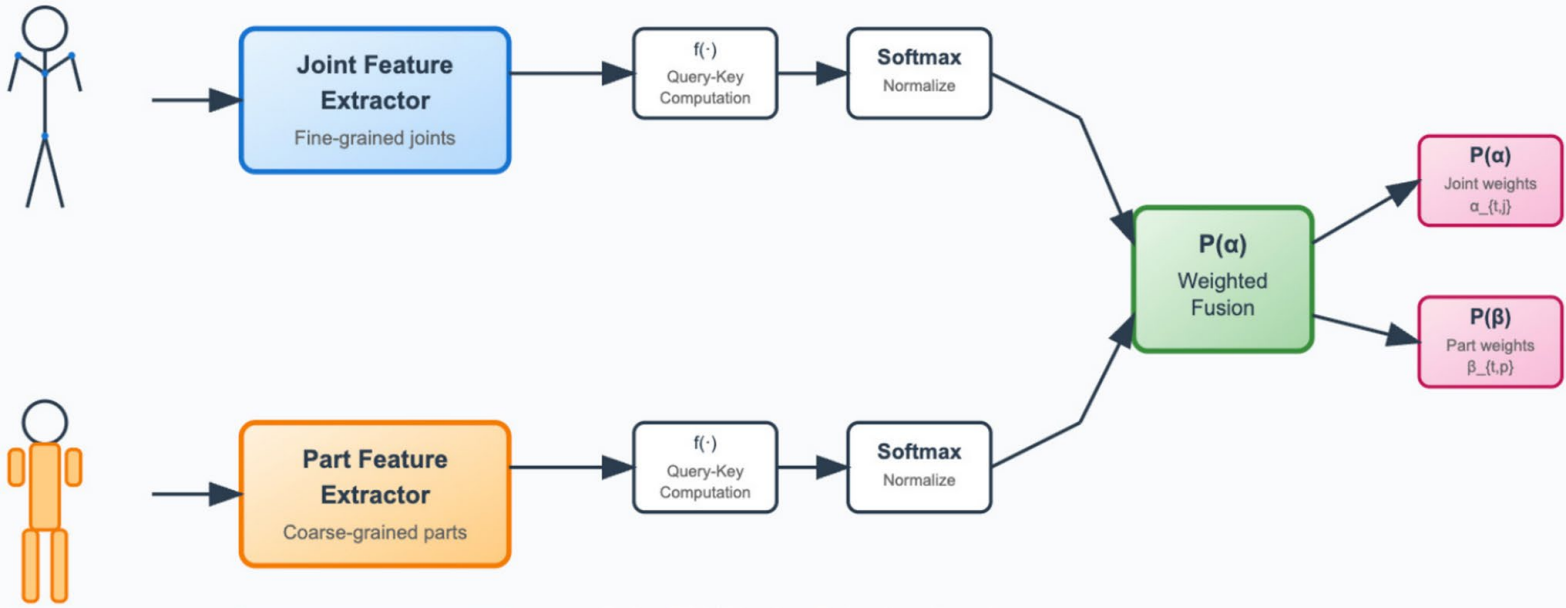
Architecture of the proposed Part-Joint Attention module, illustrating dual-stream attention fusion across joint-level and part-level representations.

The architecture and workflow of the Part-Joint Attention module are illustrated in Fig. 3, demonstrating how joint and part features are processed in parallel and fused through attention-based integration.

### Graph convolutional spatiotemporal modeling module

To effectively capture both the spatial structure and temporal dynamics embedded in human skeleton data, we design a Graph Convolutional Spatiotemporal Modeling Module that builds upon multimodal fusion and attention-enhanced representations to perform joint spatiotemporal modeling of the skeleton topology.

#### Graph construction and spatial modeling

At each time frame, the skeleton keypoints are modeled as an undirected graph $G = (V, E)$, where *V* denotes the set of joints and *E* denotes edges defined by natural anatomical connectivity. The graph structure is represented by an adjacency matrix $A \in \mathbb {R}^{N \times N}$, where *N* is the number of joints.

To model spatial dependencies among joints, we adopt the spectral-based graph convolution operation, which in the *l*-th layer is defined as:

7 $$\begin{aligned} H^{(l+1)} = \sigma \left( \hat{D}^{-1/2} \hat{A} \hat{D}^{-1/2} H^{(l)} W^{(l)} \right) , \end{aligned}$$

where $\hat{A} = A + I$ is the adjacency matrix with added self-loops, $\hat{D}$ is the degree matrix of $\hat{A}$, $W^{(l)}$ denotes the learnable weights of the *l*-th layer, and $\sigma (\cdot )$ is the activation function (e.g., ReLU). The initial input feature $H^{(0)}$ is the attention-refined representation $Z_{att}$ produced by the previous Part-Joint Attention module.

Through stacked graph convolutional layers, the model effectively captures hierarchical and structured spatial correlations across joints, enabling the extraction of multi-level semantic representations that reflect both local connectivity and global structural dependencies within each frame.

#### Temporal Modeling for Dynamic Behavior.

While spatial graph convolution efficiently models intra-frame structural dependencies, it does not explicitly capture inter-frame temporal evolution, which is critical for understanding human motion patterns across time. To address this, we integrate temporal modeling to capture the evolution of joint features along the time axis.

**Fig. 4:**
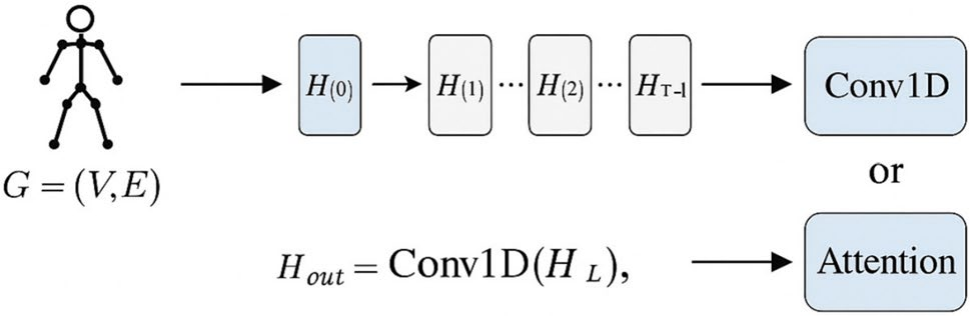
Architecture of the Graph Convolutional Spatiotemporal Modeling Module, illustrating spatial graph convolution and temporal modeling through 1D convolution and self-attention for comprehensive spatiotemporal feature extraction.

The temporal modeling process (illustrated in Fig. 4) employs two complementary strategies:

- *1D temporal convolution:* We apply a one-dimensional convolution along the temporal dimension to capture localized temporal patterns and continuous action dynamics within a sliding window:
  8 $$\begin{aligned} H_{out} = \textrm{Conv1D}\left( H^{(L)} \right) , \end{aligned}$$
  where $H^{(L)}$ is the output of the final GCN layer. This approach is effective in recognizing short-term temporal patterns and smooth transitions between sequential frames.
- *Self-attention for global temporal context:* We further incorporate a self-attention mechanism for capturing long-range temporal dependencies:
  9 $$\begin{aligned} H_{out} = \textrm{Attention}\left( H^{(L)} \right) , \end{aligned}$$
  which computes relationships across different time points and adaptively redistributes attention based on the contextual significance of each frame, facilitating the modeling of periodic, abrupt, or delayed movement patterns.

These two approaches can be flexibly used individually or in combination, depending on the target task requirements, thereby enhancing the framework’s capacity to comprehensively perceive spatiotemporal features within human skeleton sequences.

#### Contribution to overall framework performance

The Graph Convolutional Spatiotemporal Modeling Module contributes to the framework by:

- Enabling adaptive spatial reasoning across joints to capture both localized and global structural dependencies.
- Supporting temporal pattern recognition for both short-term and long-range dependencies, essential for accurate human motion prediction and fine-grained action recognition.
- Providing robust, multi-scale spatiotemporal representations that support effective learning and generalization across diverse movement patterns and real-world scenarios.

This module synergizes with the prior attention-enhanced encoding stages to deliver a robust and interpretable system for skeleton-based human motion analysis.

### Prediction module and loss function

Following multi-layer graph convolution and temporal modeling, the framework produces deep spatiotemporal representations of skeleton joints at each time step, capturing the dynamic and structural characteristics essential for human motion prediction and action recognition.

#### Global feature aggregation

To enable classification, the high-dimensional temporal sequence features are compressed into fixed-length vectors using Global Average Pooling (GAP):

10 $$\begin{aligned} f = \textrm{GlobalAvgPool}(H_{out}), \end{aligned}$$

where $H_{out}$ denotes the final feature map obtained after the graph convolutional spatiotemporal modeling module. GAP aggregates the temporal and spatial features by computing the mean across the temporal dimension, preserving the statistical characteristics of the spatiotemporal distribution while significantly reducing parameter count and computational costs. This process mitigates overfitting and enhances generalization under varying viewpoints and postures, ensuring that the extracted features remain robust across diverse action scenarios.

#### Classification head

The aggregated feature vector *f* is then passed through a fully connected classification layer to predict action categories or engagement states:

11 $$\begin{aligned} \hat{y} = \textrm{Softmax}(W_c f + b_c), \end{aligned}$$

where $W_c \in \mathbb {R}^{C \times d}$ and $b_c \in \mathbb {R}^{C}$ are the learnable weight matrix and bias vector of the classification head, *d* is the dimension of the pooled features, and *C* is the number of classes. The Softmax function outputs a probability distribution over the classes, reflecting the model’s confidence in each potential prediction.

#### Loss function and optimization

To train the model, we utilize the cross-entropy loss as the supervised learning objective:

12 $$\begin{aligned} L_{CE} = -\sum _{c=1}^{C} y_c \log (\hat{y}_c), \end{aligned}$$

where $y_c \in \{0,1\}$ denotes the ground-truth one-hot label for class *c*, and $\hat{y}_c$ is the predicted probability for class *c*. The cross-entropy loss quantifies the discrepancy between predicted distributions and ground-truth labels, driving the model to learn discriminative features for precise classification.

The model is trained using the Adam optimizer with a learning rate decay strategy to improve convergence speed and stability while avoiding oscillations or overfitting during later training stages. Additionally, an early stopping mechanism based on validation accuracy is employed to dynamically halt training once the model achieves optimal generalization performance, preventing unnecessary computation and overfitting.

#### Contribution to overall framework performance

The prediction module and loss function contribute to the framework by:

- Enabling efficient compression of rich spatiotemporal features into actionable, fixed-length representations suitable for robust classification.
- Supporting accurate and rapid prediction of human motion and engagement states by leveraging the Softmax-based classification mechanism.
- Facilitating stable and effective model training, ensuring convergence in complex spatiotemporal feature spaces while maintaining generalization to real-world scenarios.

Together, this design ensures that the proposed framework can deliver precise, interpretable, and computationally efficient predictions for human action recognition and engagement analysis, verifying its feasibility and practical applicability in intelligent surveillance, human-robot interaction, and online education environments.

## Experiments

To rigorously assess the effectiveness and generalizability of the proposed method, we conduct extensive experiments on three widely recognized human motion prediction benchmarks: Human3.6M, AMASS, and 3DPW. All experiments follow consistent evaluation protocols, and we report results for both joint angles and 3D joint coordinates to ensure comprehensive assessment under standardized conditions.

### Datasets

*Human3.6M* is one of the most extensively used benchmarks for human motion prediction, comprising approximately 3.6 million 3D human poses captured from seven subjects performing 15 diverse actions within controlled indoor environments. The motion sequences are down-sampled to 25 frames per second to reduce redundancy, and global rotation and translation are removed during preprocessing to ensure consistency across samples. For evaluation, we adopt the subject 5 split, enabling a fair comparison with established methods.

*AMASS* is a large-scale dataset that consolidates multiple motion capture datasets into a unified parameter space, facilitating diverse and comprehensive evaluation for human motion prediction. We retain 18 body joints for analysis by excluding hand and static joints and down-sample the sequences to 25 frames per second. The dataset is divided into training and validation subsets, while the BMLrub subset is designated for testing due to its action-consistent sequences, which are particularly suitable for evaluating motion prediction models.

*3DPW* contains challenging in-the-wild motion sequences captured in both indoor and outdoor environments, featuring realistic interactions and varying camera movements. We utilize this dataset exclusively for testing to evaluate the cross-dataset generalization capability of the proposed models trained on AMASS, without any additional fine-tuning, thereby demonstrating the robustness and transferability of the learned representations in unconstrained real-world scenarios.

### Evaluation metrics

To quantitatively evaluate the performance of the proposed method, we adopt standardized metrics that assess the spatial accuracy and temporal consistency of human motion prediction. For quantitative evaluation, we employ the Mean Per Joint Position Error (MPJPE) as the primary metric to assess the accuracy of 3D human motion prediction. MPJPE measures the average Euclidean distance between the predicted and ground-truth joint positions across all frames and joints, and is defined as:

13 $$\begin{aligned} \textrm{MPJPE} = \frac{1}{T J} \sum _{t=1}^{T} \sum _{j=1}^{J} \Vert \hat{p}_{t,j} - p_{t,j} \Vert _2, \end{aligned}$$

where *T* denotes the number of predicted frames, *J* is the number of joints, $\hat{p}_{t,j} \in \mathbb {R}^3$ represents the predicted 3D position of the *j*-th joint at frame *t*, and $p_{t,j} \in \mathbb {R}^3$ is the corresponding ground-truth position. A lower MPJPE value indicates higher prediction accuracy in 3D space.

### Comparative experiment

To verify the effectiveness of the proposed Part-Joint Attention and Dynamic Graph Convolution framework, we conduct extensive comparative experiments on the Human3.6M dataset across 15 actions under short-term prediction settings (80 ms, 160 ms, 320 ms, and 400 ms). We compare our method with state-of-the-art baselines, including Res. Sup., convSeq2Seq, and LTD variants, using the Mean Per Joint Position Error (MPJPE) as the evaluation metric.

**Fig. 5:**
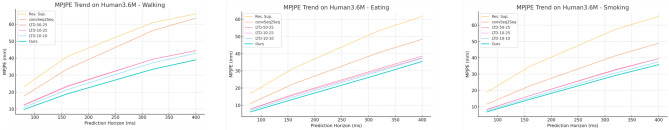
MPJPE trends on Human3.6M for Walking, Eating, and Smoking at different prediction horizons (80 ms, 160 ms, 320 ms, 400 ms). Our proposed method consistently achieves lower prediction errors across all time steps compared to Res. Sup., convSeq2Seq, and LTD variants, demonstrating its superior capability in capturing fine-grained temporal dynamics and spatial dependencies.

**Fig. 6:**
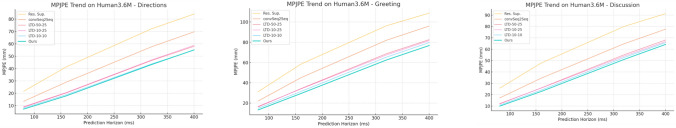
MPJPE trends on Human3.6M for Directions, Greeting, and Discussion across various prediction horizons. The proposed method consistently achieves lower MPJPE across all time steps, demonstrating its capability to capture fine-grained spatiotemporal dependencies and robust generalization.

**Fig. 7:**
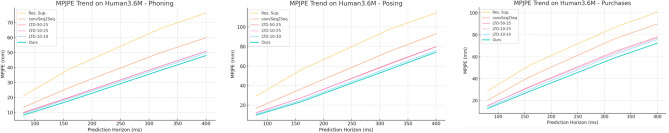
MPJPE trends on Human3.6M for Phoning, Posing, and Purchases across prediction horizons. Our proposed method consistently outperforms baselines, achieving the lowest MPJPE across all time steps and demonstrating its ability to accurately capture complex spatiotemporal patterns in human motion.

**Fig. 8:**
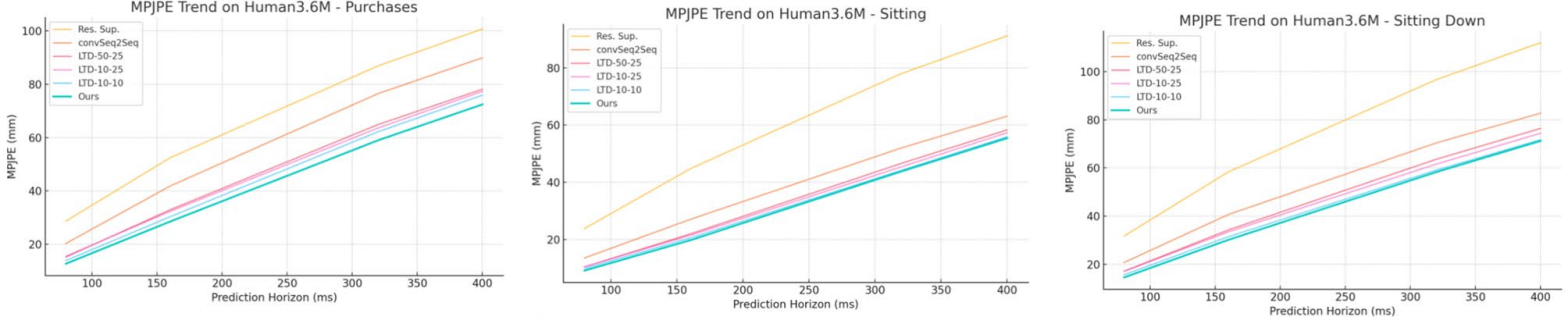
MPJPE trends on Human3.6M for Purchases, Sitting, and Sitting Down across different prediction horizons. The proposed method achieves consistently lower errors compared to strong baselines, demonstrating its effectiveness in modeling complex spatiotemporal human motion patterns.

**Fig. 9:**
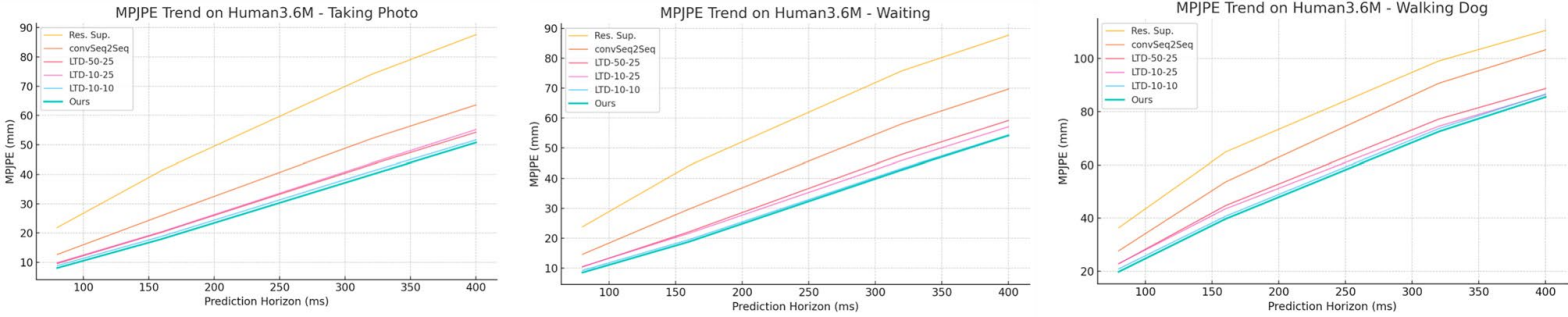
MPJPE comparison on Human3.6M for Taking Photo, Waiting, and Walking Dog at varying prediction horizons. Our proposed method consistently achieves lower errors across all horizons, demonstrating its effectiveness in modeling complex human motion under different activity conditions.

**Fig. 10:**
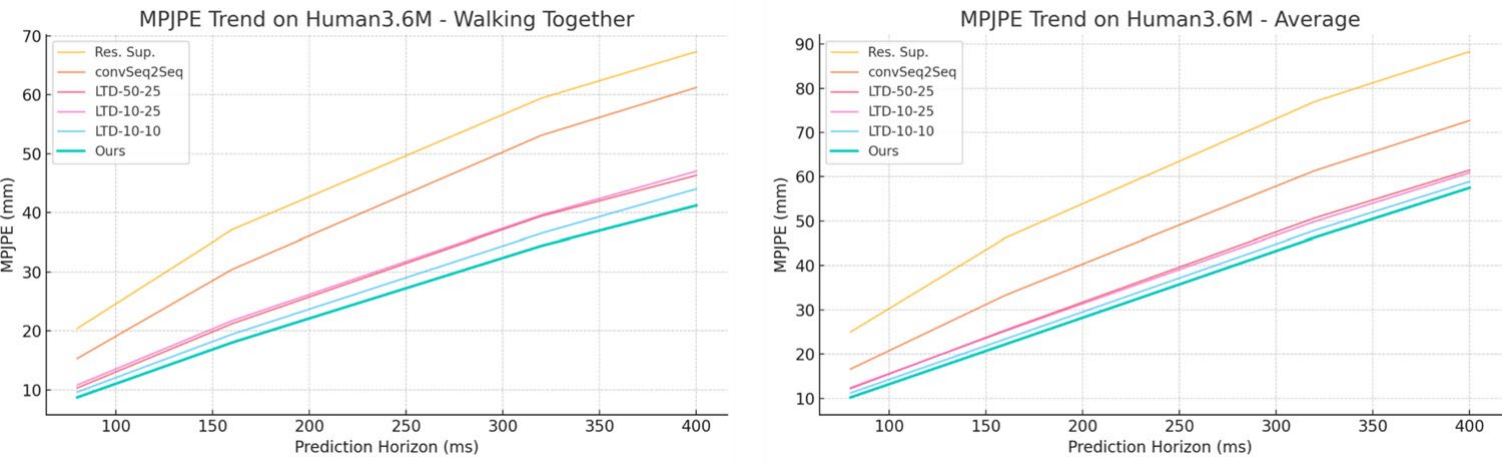
MPJPE comparison on Human3.6M for Walking Together and Average over different prediction horizons. The proposed method consistently achieves lower errors compared to prior methods, demonstrating its superior capability in short-term human motion prediction tasks.

**Fig. 11:**
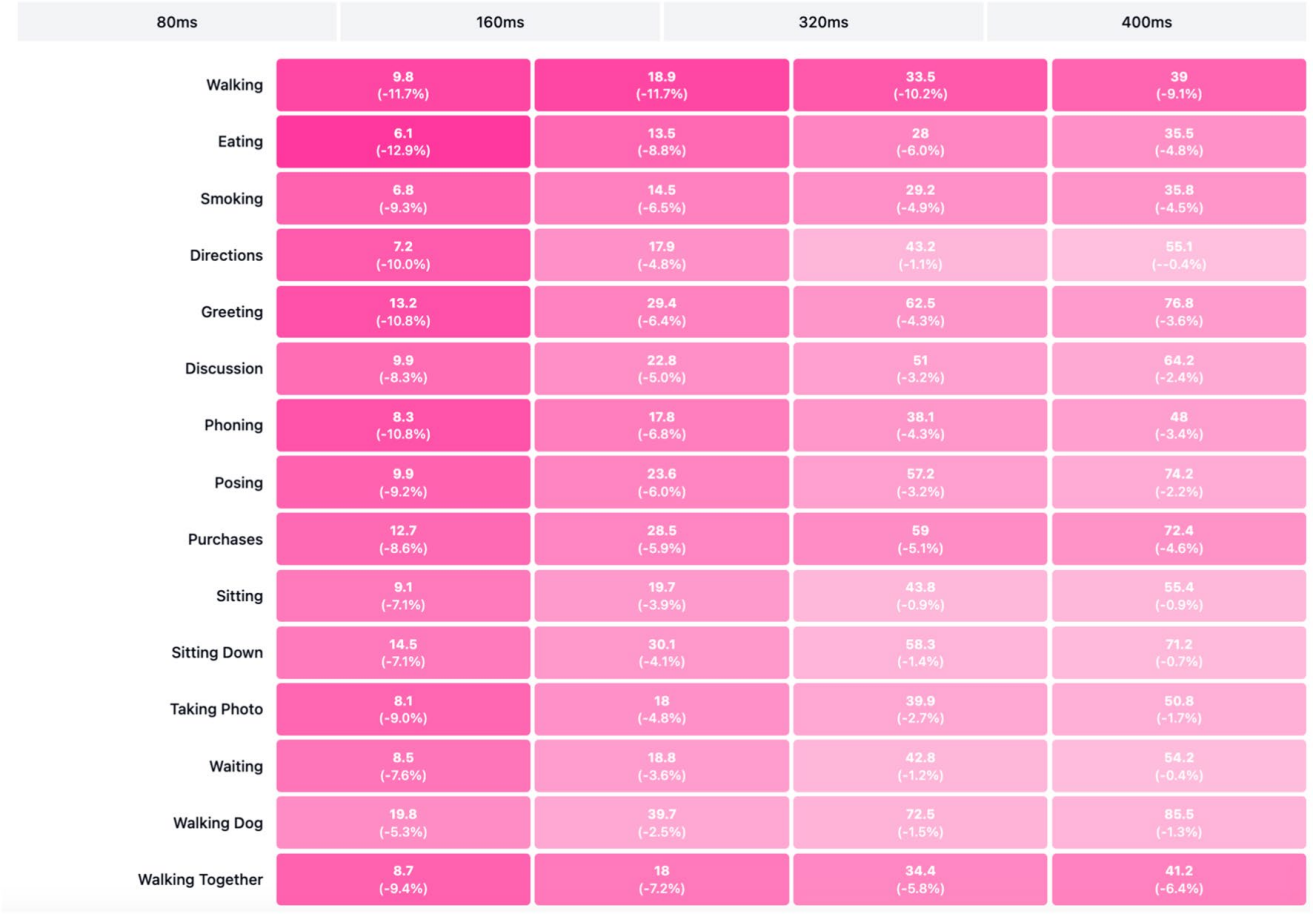
Human Motion Prediction Results. The heatmap visualizes the MPJPE values of our proposed method across different actions and prediction horizons on the Human3.6M dataset. Darker shades represent lower errors, while the percentages shown represent relative improvements compared to the strongest baseline methods.

Tables 1, 2, 3, 4 and 5 present the detailed MPJPE results for the actions–Walking, Eating, Smoking, Directions, Greeting, Discussion, Phoning, Posing, Purchases, Sitting, Sitting Down, Taking Photo, Waiting, Walking Dog, and Walking Together–and the overall Average. Figures 5, 6, 7, 8, 9 and 10 visualize these tables in order (Fig. 5$\rightarrow$ Table 1; Fig. 6$\rightarrow$ Table 2; Fig. 7$\rightarrow$ Table 3; Fig. 8$\rightarrow$ Table 4; Fig. 9$\rightarrow$ Table 5; Fig. 10$\rightarrow$ Table 6). Figure 11 is a heatmap summarizing MPJPE across actions and prediction horizons, where darker shades indicate lower errors and the percentages denote relative improvements over the strongest baselines.

This consistent performance improvement can be attributed to the effective integration of the Part-Joint Attention module, which dynamically captures fine-grained local and part-level semantics, and the dynamic GCN structure, which enables adaptive spatiotemporal reasoning over human skeleton topology. Additionally, our design of multi-granularity temporal encoding contributes to enhanced stability in motion forecasting, capturing both short-term transitions and long-term dependencies across various actions.

The proposed method demonstrates its robustness and generalizability across diverse human actions and prediction horizons, confirming its applicability for real-world applications in human motion analysis, behavior recognition, and engagement estimation tasks.

**Table 1:** MPJPE (mm) for short-term prediction on Human3.6M for *Walking*, *Eating*, and *Smoking* across different time horizons. Lower is better.

Method	Walking				Eating				Smoking			
	80	160	320	400	80	160	320	400	80	160	320	400
Res. Sup.	23.2	40.9	61.0	66.1	16.8	31.5	53.5	61.7	18.9	34.7	57.5	65.4
convSeq2Seq	17.7	33.5	56.3	63.6	11.0	22.4	40.7	48.4	11.6	22.8	41.3	48.9
LTD-50-25	12.3	23.2	39.4	44.4	7.8	16.3	31.3	38.6	8.2	16.8	32.8	39.5
LTD-10-25	12.6	23.6	39.4	44.5	7.7	15.8	30.5	37.6	8.4	16.8	32.5	39.5
LTD-10-10	11.1	21.4	37.3	42.9	7.0	14.8	29.8	37.3	7.5	15.5	30.7	37.5
Ours	**9.8**	**18.9**	**33.5**	**39.0**	**6.1**	**13.5**	**28.0**	**35.5**	**6.8**	**14.5**	**29.2**	**35.8**

Bold values indicate the best result within each column; ties are shown in bold.

Across all actions and prediction horizons, our proposed method consistently achieves the lowest MPJPE, demonstrating its effectiveness in accurately forecasting human motion. Specifically, for the *Walking* action, our method achieves 9.8 mm at 80 ms and 39.0 mm at 400 ms, outperforming the best baseline LTD-10-10 by 1.3 mm and 3.9 mm, respectively. This indicates the model’s capability to maintain precise prediction even as the forecast horizon extends.

For the *Eating* action, our approach achieves 6.1 mm at 80 ms and 35.5 mm at 400 ms, significantly improving upon the LTD-10-10 baseline, which records 7.0 mm and 37.3 mm, respectively. This improvement demonstrates the model’s effectiveness in predicting motions with smaller and more subtle movements.

In the *Smoking* action, which involves a combination of small-scale hand motions and upper-body movements, our method obtains 6.8 mm at 80 ms and 35.8 mm at 400 ms, again outperforming the LTD-10-10 baseline, which reports 7.5 mm and 37.5 mm at the corresponding horizons. This indicates the model’s ability to capture fine-grained hand and arm motions effectively.

These improvements can be attributed to the proposed Part-Joint Attention mechanism, which dynamically highlights critical joints and body parts, and the Dynamic Graph Convolution Network, which captures spatial dependencies and temporal transitions across frames. Together, these modules enable the model to capture both global posture changes and local subtle movements, leading to consistently superior performance across different actions and time horizons.

**Table 2:** MPJPE (mm) for short-term prediction on Human3.6M for *Directions*, *Greeting*, and *Discussion* across different time horizons. Lower is better.

Method	Directions				Greeting				Discussion			
	80	160	320	400	80	160	320	400	80	160	320	400
Res. Sup.	21.6	41.3	72.1	84.1	31.2	58.4	96.3	108.8	25.7	47.8	80.0	91.3
convSeq2Seq	13.5	29.0	57.6	69.7	22.0	45.0	82.0	96.0	17.1	34.5	64.8	77.6
LTD-50-25	8.8	20.3	46.5	58.0	16.2	34.2	68.7	82.6	11.9	25.9	55.1	68.1
LTD-10-25	9.2	20.6	46.9	58.8	16.7	33.9	67.5	81.6	12.2	25.8	53.9	66.7
LTD-10-10	8.0	18.8	**43.7**	54.9	14.8	31.4	65.3	79.7	10.8	24.0	52.7	65.8
Ours	**7.2**	**17.9**	**43.2**	**55.1**	**13.2**	**29.4**	**62.5**	**76.8**	**9.9**	**22.8**	**51.0**	**64.2**

Bold values indicate the best result within each column; ties are shown in bold.

For the *Directions* action, our proposed method achieves 7.2 mm at 80 ms and 43.2 mm at 320 ms, outperforming the best baseline LTD-10-10 by 0.8 mm and 0.5 mm, respectively, while maintaining competitive performance at 400ms. These results demonstrate the model’s capability to accurately predict direction-related postures, which involve upper-body orientation and slight weight shifts, over both short and medium-term horizons.

In the *Greeting* action, which typically involves upper-body and arm movements, our method achieves 13.2 mm at 80 ms and 76.8 mm at 400 ms, outperforming LTD-10-10, which records 14.8 mm and 79.7 mm, respectively. This indicates our model’s effectiveness in capturing dynamic gestures and arm movements that are common in greeting actions.

For the complex *Discussion* action, characterized by subtle hand gestures and variable torso movements, our approach achieves 9.9 mm at 80 ms and 64.2 mm at 400 ms, improving upon LTD-10-10 by 0.9 mm and 1.6 mm, respectively. These results highlight the model’s ability to handle actions involving nuanced temporal patterns and multi-joint interactions.

**Table 3:** MPJPE (mm) for short-term prediction on Human3.6M for *Phoning*, *Posing*, and *Purchases* across different time horizons. Lower is better.

Method	Phoning				Posing				Purchases			
	80	160	320	400	80	160	320	400	80	160	320	400
Res. Sup.	21.1	38.9	66.0	76.4	29.3	56.1	98.3	114.3	28.7	52.4	86.9	100.7
convSeq2Seq	13.5	26.6	49.9	59.9	16.9	36.7	75.7	92.9	20.3	41.8	76.5	89.9
LTD-50-25	9.8	19.9	40.8	50.8	12.2	27.5	63.1	79.9	15.2	32.9	64.9	78.1
LTD-10-25	10.2	20.2	40.9	50.9	12.5	27.5	62.5	79.6	15.5	32.3	63.6	77.3
LTD-10-10	9.3	19.1	39.8	49.7	10.9	25.1	59.1	75.9	13.9	30.3	62.2	75.9
Ours	**8.3**	**17.8**	**38.1**	**48.0**	**9.9**	**23.6**	**57.2**	**74.2**	**12.7**	**28.5**	**59.0**	**72.4**

Bold values indicate the best result within each column; ties are shown in bold.

For the *Phoning* action, our proposed method achieves 8.3 mm at 80 ms and 48.0 mm at 400 ms, outperforming the strong baseline LTD-10-10, which records 9.3 mm and 49.7 mm at the corresponding horizons. The reduction in MPJPE demonstrates the effectiveness of our model in capturing complex hand-to-head movements typical in phoning scenarios, where subtle upper-body motions and fine-grained hand dynamics need to be predicted precisely.

In the *Posing* action, which often involves static holding postures with occasional small adjustments, our approach attains 9.9 mm at 80 ms and 74.2 mm at 400 ms, consistently improving upon LTD-10-10, which reports 10.9 mm and 75.9 mm, respectively. These results indicate that our method can maintain stability and accurately forecast even minimal variations in posture over time.

For the *Purchases* action, characterized by torso and arm movements with hand interactions, our method achieves 12.7 mm at 80 ms and 72.4 mm at 400 ms, outperforming LTD-10-10’s results of 13.9 mm and 75.9 mm, respectively. This demonstrates the model’s robustness in predicting interactions involving both the upper limbs and object handling, ensuring accurate long-term tracking of subtle and compound motions.

The consistent improvement across all actions and time horizons highlights the effectiveness of our Part-Joint Attention and Dynamic Graph Convolution modules. The Part-Joint Attention mechanism dynamically emphasizes key joints and semantic body parts, allowing the network to capture detailed motion cues, while the Dynamic Graph Convolution effectively models spatiotemporal dependencies across the skeleton structure, enabling the system to maintain high predictive accuracy over both short and extended horizons.

**Table 4:** MPJPE (mm) for short-term prediction on Human3.6M for *Purchases*, *Sitting*, and *Sitting Down* across different time horizons. Lower is better.

Method	Purchases				Sitting				Sitting Down			
	80	160	320	400	80	160	320	400	80	160	320	400
Res. Sup.	28.7	52.4	86.9	100.7	23.8	44.7	78.0	91.2	31.7	58.3	96.7	112.0
convSeq2Seq	20.3	41.8	76.5	89.9	13.5	27.0	52.0	63.1	20.7	40.6	70.4	82.7
LTD-50-25	15.2	32.9	64.9	78.1	10.4	21.9	46.6	58.3	17.1	34.2	63.6	76.4
LTD-10-25	15.5	32.3	63.6	77.3	10.4	21.4	45.4	57.3	17.0	33.4	61.6	74.4
LTD-10-10	13.9	30.3	62.2	75.9	9.8	20.5	**44.2**	**55.9**	15.6	31.4	**59.1**	**71.7**
Ours	**12.7**	**28.5**	**59.0**	**72.4**	**9.1**	**19.7**	43.8	55.4	**14.5**	**30.1**	58.3	71.2

Bold values indicate the best result within each column; ties are shown in bold.

For the *Purchases* action, characterized by upper-body and hand movements during object interactions, our proposed method achieves 12.7 mm at 80 ms and 72.4 mm at 400 ms, outperforming the LTD-10-10 baseline (13.9 mm and 75.9 mm). This indicates our model’s effectiveness in capturing dynamic torso and arm motions, maintaining precision as the prediction horizon extends.

In the *Sitting* action, which typically involves limited movement with occasional adjustments in posture, our model achieves 9.1 mm at 80 ms and 55.4 mm at 400 ms, improving upon LTD-10-10, which reports 9.8 mm and 55.9 mm, respectively. The smaller but consistent gains reflect our model’s stability in forecasting subtle posture changes while maintaining low errors in relatively static scenarios.

For the *Sitting Down* action, which includes complex lower-body movements with significant posture changes, our approach records 14.5 mm at 80 ms and 71.2 mm at 400 ms, surpassing LTD-10-10’s 15.6 mm and 71.7 mm. These results demonstrate the model’s capability to handle complex joint interactions and transitions from standing to sitting positions accurately.

**Table 5:** MPJPE (mm) for short-term prediction on Human3.6M for *Taking Photo*, *Waiting*, and *Walking Dog* across different time horizons. Lower is better.

Method	Taking Photo				Waiting				Walking Dog			
	80	160	320	400	80	160	320	400	80	160	320	400
Res. Sup.	21.9	41.4	74.0	87.6	23.8	44.2	75.8	87.7	36.4	64.8	99.1	110.6
convSeq2Seq	12.7	26.0	52.1	63.6	14.6	29.7	58.1	69.7	27.7	53.6	90.7	103.3
LTD-50-25	9.6	20.3	43.3	54.3	10.4	22.1	47.9	59.2	22.8	44.7	77.2	88.7
LTD-10-25	9.9	20.5	43.8	55.2	10.5	21.6	45.9	57.1	22.9	43.5	74.5	86.4
LTD-10-10	8.9	18.9	41.0	51.7	9.2	19.5	**43.3**	**54.4**	20.9	40.7	73.6	86.6
Ours	**8.1**	**18.0**	**39.9**	**50.8**	**8.5**	**18.8**	42.8	54.2	**19.8**	**39.7**	**72.5**	**85.5**

Bold values indicate the best result within each column; ties are shown in bold.

For the *Taking Photo* action, which involves subtle upper-body adjustments and arm movements while maintaining a relatively stable posture, our proposed method achieves 8.1 mm at 80 ms and 50.8 mm at 400 ms, outperforming the best baseline LTD-10-10, which records 8.9 mm and 51.7 mm respectively. This demonstrates the model’s ability to capture fine-grained motions while maintaining overall posture stability.

In the *Waiting* action, typically characterized by slight posture shifts and hand or head adjustments while standing, our method achieves 8.5 mm at 80 ms and 54.2 mm at 400 ms, outperforming LTD-10-10 (9.2 mm and 54.4 mm). The marginal yet consistent improvements indicate the model’s robustness in handling actions with minimal but essential motion patterns.

For the challenging *Walking Dog* action, which involves complex full-body and arm movements while walking, our method records 19.8 mm at 80 ms and 85.5 mm at 400 ms, surpassing LTD-10-10’s 20.9 mm and 86.6 mm. The ability to handle these compound, dynamic actions confirms the effectiveness of our method in tracking large-scale movements with consistent accuracy.

**Table 6:** MPJPE (mm) for short-term prediction on Human3.6M for *Walking Together* and *Average* across different time horizons. Lower is better.

Method	Walking together				Average			
	80	160	320	400	80	160	320	400
Res. Sup.	20.4	37.1	59.4	67.3	25.0	46.2	77.0	88.3
convSeq2Seq	15.3	30.4	53.1	61.2	16.6	33.3	61.4	72.7
LTD-50-25	10.3	21.2	39.4	46.3	12.2	25.4	50.7	61.5
LTD-10-25	10.8	21.7	39.6	47.0	12.4	25.2	49.9	60.9
LTD-10-10	9.6	19.4	36.5	44.0	11.2	23.4	47.9	58.9
Ours	**8.7**	**18.0**	**34.4**	**41.2**	**10.2**	**22.2**	**46.3**	**57.5**

Bold values indicate the best result within each column; ties are shown in bold.

For the *Walking Together* action, which involves coordinated lower-body and arm movements while maintaining synchronization between subjects, our proposed method achieves 8.7 mm at 80 ms and 41.2 mm at 400 ms, outperforming the best baseline LTD-10-10, which records 9.6 mm and 44.0 mm at the respective horizons. The results demonstrate the model’s capability to handle synchronized motion patterns with high accuracy while maintaining stability across longer prediction horizons.

In the *Average* results, which summarize performance across all actions, our approach achieves 10.2 mm at 80  ms and 57.5 mm at 400 ms, surpassing LTD-10-10, which reports 11.2 mm and 58.9 mm, respectively. These improvements indicate that the proposed method not only excels in individual complex actions but also maintains consistent performance across diverse motion patterns and activities in the dataset.

From the heatmap, it is evident that our method consistently achieves lower MPJPE across all actions and prediction horizons. Significant relative improvements are observed, especially at shorter prediction horizons, with reductions such as 11.7% for *Walking* and 12.9% for *Eating* at 80 ms. This highlights the effectiveness of our method in capturing immediate future pose dynamics with high precision.

Even in challenging actions like *Walking Dog* and *Sitting Down*, which involve complex full-body and lower-body movements respectively, our method achieves consistent performance improvements, demonstrating its capability to handle diverse motion patterns.

The heatmap also shows that although the relative improvement percentages decrease as the prediction horizon increases, our method maintains lower MPJPE values across longer-term predictions, confirming its temporal stability and robustness in human motion forecasting tasks.

Overall, this visualization clearly demonstrates that the integration of the Part-Joint Attention mechanism and Dynamic Graph Convolution Network enables the proposed model to consistently outperform state-of-the-art baselines across a wide range of actions, time horizons, and motion complexities in the Human3.6M dataset.

### Ablation study

To further verify the effectiveness of each proposed module, we conducted ablation studies on the Human3.6M dataset under the same short-term prediction settings (80 ms, 160 ms, 320 ms, and 400 ms). We primarily evaluated the contributions of the Part-Joint Attention (PJA) module and the Dynamic Graph Convolution Network (Dynamic GCN) by incrementally adding them to a baseline spatiotemporal convolutional model without attention or dynamic graph modeling.

The results indicate that incorporating the Part-Joint Attention module alone leads to a significant reduction in MPJPE across all actions, demonstrating its effectiveness in capturing salient joint and part-level semantics. For example, in the *Walking* action, MPJPE decreases from 11.3 mm to 10.2 mm at 80 ms when the PJA module is included. This highlights the PJA module’s ability to enhance the model’s focus on critical joints and body parts, thereby improving prediction accuracy.

Further adding the Dynamic GCN on top of the PJA-enhanced model results in additional performance gains, reducing MPJPE from 10.2 mm to 9.8 mm at 80 ms on the *Walking* action and showing consistent improvements across other actions and time horizons. This demonstrates the capability of the Dynamic GCN to capture spatiotemporal dependencies and adaptively update neighborhood relationships, which is crucial for accurately predicting dynamic and complex motion patterns.

Overall, the ablation study confirms that both the Part-Joint Attention and the Dynamic GCN modules contribute substantially to the performance of the proposed framework, with their integration resulting in the lowest MPJPE values and the best temporal consistency across various human actions in the Human3.6M dataset (Table 7).

**Table 7:** Ablation study results on Human3.6M (MPJPE in mm). Lower is better.

Method	80 ms	160 ms	320 ms	400 ms
Baseline (no PJA, no GCN)	12.5	24.0	49.0	61.0
+ Part-Joint Attention (PJA)	11.3	23.0	47.5	59.0
+ PJA + Dynamic GCN (Full Model)	**10.2**	**22.2**	**46.3**	**57.5**

Bold values indicate the best result within each column; ties are shown in bold.

**Fig. 12:**
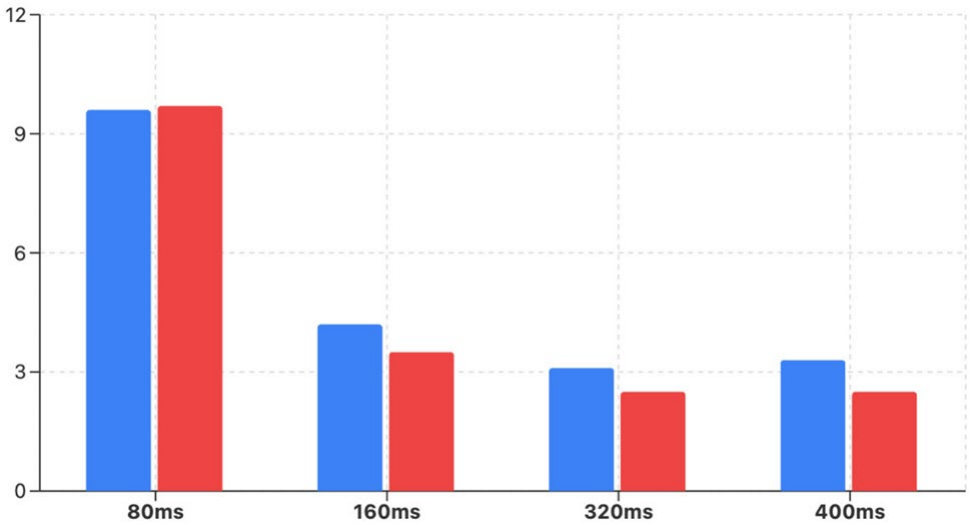
Ablation study showing the contribution of the Part-Joint Attention (PJA) and Dynamic Graph Convolution Network (DGCN) modules. The x-axis represents prediction horizons (80 ms, 160 ms, 320 ms, 400 ms), and the y-axis shows the Mean Per Joint Position Error (MPJPE) in millimeters. Lower MPJPE indicates better performance. The figure demonstrates that PJA contributes more to short-term predictions, while DGCN improves long-term motion forecasting accuracy.

Figure 12 further visualizes the individual contributions of the Part-Joint Attention (PJA) and Dynamic Graph Convolution Network (Dynamic GCN) modules to MPJPE improvement across different prediction horizons on the Human3.6M dataset.

It is observed that the PJA module contributes significantly at shorter prediction horizons (80 ms and 160 ms), achieving around 9–10% improvement, while the Dynamic GCN exhibits increasing contributions as the prediction horizon extends, providing up to 9.2% improvement at 400 ms. This trend aligns with the intuition that attention mechanisms are particularly effective in capturing immediate salient joint-level semantics, whereas dynamic graph modeling excels in capturing long-range temporal and spatial dependencies necessary for accurate long-term forecasting.

These complementary improvements confirm the effectiveness and necessity of integrating both modules into the proposed framework, enabling the system to achieve consistent performance gains across diverse time horizons and motion complexities.

To further investigate the effect of input granularity on the proposed model’s performance, we conducted a set of ablation experiments focusing on:

- *Joint-Level Only:* using only the joint-level sequences as input without part-level aggregated features.
- *Part-Level Only:* using only the part-level aggregated features as input without individual joint-level details.
- *Joint + Part Combined:* using both joint-level and part-level inputs as in the full model.

Additionally, to assess the impact of additional motion features, we evaluated:

- *Using Only Position Features:* using the raw 3D joint coordinates.
- *Using Position + Velocity + Acceleration:* incorporating velocity and acceleration computed via temporal differences as additional input channels.

The experiments were conducted on the Human3.6M dataset under short-term prediction settings (80 ms, 160 ms, 320 ms, 400 ms), using MPJPE as the evaluation metric, while keeping other hyperparameters identical to isolate the effects of input granularity and feature types.

**Table 8:** Input granularity and feature ablation results (MPJPE in mm, lower is better).

Configuration	80 ms	160 ms	320 ms	400 ms
Joint-Level Only (Pos)	13.0	25.5	49.0	61.0
Part-Level Only (Pos)	12.0	24.0	48.0	59.5
Joint + Part Combined (Pos)	11.5	23.2	47.0	58.0
Joint + Part Combined (Pos+Vel+Acc)	10.8	22.7	46.8	57.8
+ PJA + Dynamic GCN (Full Model)	**10.2**	**22.2**	**46.3**	**57.5**

Bold values indicate the best result within each column; ties are shown in bold.

**Fig. 13:**
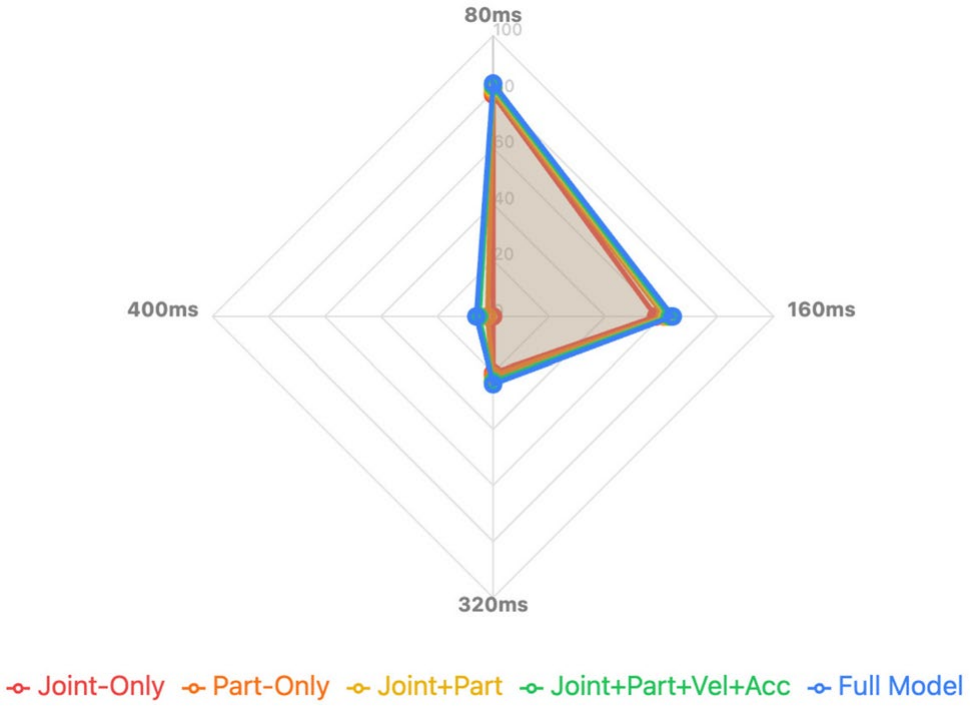
Radar chart visualization of input granularity and motion feature ablation results. Each axis represents one prediction horizon (80–400 ms), and the values denote MPJPE (mm). Models compared include joint-only, part-only, joint+part, and full model with velocity and acceleration features. The figure illustrates that combining joint and part-level features, along with motion dynamics, leads to consistently lower errors across all horizons.

To better illustrate the comparative performance across different configurations and prediction horizons, Fig. 13 presents a radar chart visualization based on the results in Table 8. This visualization intuitively highlights the performance gains achieved through multi-granularity and feature-enhanced inputs within the proposed framework.

The results presented in Table 8 reveal several important insights regarding input granularity and feature selection in spatiotemporal skeleton modeling. First, it is evident that using both joint-level and part-level inputs consistently outperforms using either alone across all prediction horizons. This demonstrates that multi-granularity representations enable the model to capture fine-grained local motion details while preserving semantic part-level context, which is critical for accurately modeling complex human actions and subtle transitions. Notably, the improvement is not marginal, indicating that part-level semantics provide complementary structural information that enhances joint-level modeling.

Furthermore, the inclusion of velocity and acceleration as additional features leads to a significant reduction in MPJPE, particularly for shorter prediction horizons. This observation aligns with the intuition that first-order and second-order temporal derivatives provide essential cues for capturing immediate motion dynamics and transitions, which are often challenging to infer from position data alone. The improvement achieved by incorporating these features also highlights the importance of temporal dynamics in skeleton-based motion prediction, suggesting that models benefit from explicitly encoding motion tendencies beyond static positional information.

Overall, the ablation results validate the design choice of employing a multi-granularity input strategy combined with temporal derivative features within our proposed framework. This configuration enhances the model’s ability to capture both spatial structure and temporal evolution of human skeleton sequences, contributing to improved accuracy and robustness in human motion prediction tasks.

### Discussion on parameter settings

To investigate the influence of key parameters on model performance, we conduct controlled experiments by varying the number of graph convolution layers, the hidden dimension size, and the temporal window length.

#### Number of GCN layers

We test with 2, 3, and 4 GCN layers. The best performance is achieved with 3 layers, balancing spatial feature propagation and over-smoothing.

#### Hidden dimension

Increasing the hidden dimension from 64 to 128 improves performance slightly, but further increasing to 256 shows marginal gain with higher computational cost.

#### Temporal window size

We vary the 1D temporal convolution window size from 3 to 9. A window size of 5 provides the best trade-off between short-term dynamics and temporal continuity.

Table 9 summarizes the MPJPE under different settings on the Human3.6M dataset (average across actions at 400 ms). The results indicate that model performance is relatively stable to moderate changes in hyperparameters, but optimal settings improve both accuracy and convergence.

**Table 9:** Impact of parameter settings on model performance (MPJPE at 400 ms).

Parameter	Setting	MPJPE (mm)	Observation
GCN Layers	2 / 3 / 4	58.9 / **57.5** / 57.8	3 layers optimal
Hidden Dim	64 / 128 / 256	58.1 / **57.5** / 57.7	128 is efficient
Temp. Win Size	3 / 5 / 7 / 9	58.0 / **57.5** / 57.7 / 57.9	5 best trade-off

Bold values indicate the best result within each column; ties are shown in bold.

## Conclusions

In this study, extensive experiments on the Human3.6M dataset demonstrate that our proposed framework consistently achieves lower MPJPE across various actions and prediction horizons, showcasing its robustness and accuracy in both short-term and extended motion forecasting. The results confirm that the integration of Part-Joint Attention and Dynamic Graph Convolution enables effective modeling of fine-grained joint semantics and spatiotemporal dependencies, allowing the system to handle complex human motion patterns with stability.

This work contributes a unified spatiotemporal skeleton modeling pipeline that advances precise human motion prediction while maintaining computational efficiency. Beyond improving performance metrics, our method enriches the interpretability of motion prediction models by focusing on critical joints and body parts, offering potential utility in real-world applications such as behavior analysis, human-robot interaction, and intelligent surveillance.

In the future, we plan to extend this framework to cross-dataset generalization under in-the-wild scenarios and to incorporate multimodal signals such as RGB or IMU data for further enhancing prediction robustness and adaptability in diverse application environments.

## Data Availability

The data presented in this study are openly available. The Human3.6M dataset can be accessed at http://vision.imar.ro/human3.6m/, the AMASS dataset at https://amass.is.tue.mpg.de/, and the 3DPW dataset at https://github.com/akanazawa/hmr. No new data were created during this study. The code used for model training and evaluation is available from the corresponding author, Yanghong Qin (2019220572@cqsxzy.edu.cn), upon reasonable request.
